# Association of Blood MicroRNA Expression and Polymorphisms with Cognitive and Biomarker Changes in Older Adults

**DOI:** 10.14283/jpad.2023.99

**Published:** 2023-09-06

**Authors:** A. Sadlon, P. Takousis, E. Evangelou, I. Prokopenko, P. Alexopoulos, C.-M. Udeh-Momoh, G. Price, L. Middleton, Robert Perneczky, Alzheimer’s Disease Neuroimaging Initiative

**Affiliations:** 1 Ageing Epidemiology (AGE) Research Unit, School of Public Health, Imperial College London, London, UK; 2 Department of Epidemiology and Biostatistics, School of Public Health, Imperial College London, London, UK; 3 Department of Hygiene and Epidemiology, University of Ioannina Medical School, Ioannina, Greece; 4 Department of Clinical & Experimental Medicine, University of Surrey, Guildford, UK; 5 UMR 8199–EGID, Institut Pasteur de Lille, CNRS, University of Lille, Lille, France; 6 Department of Psychiatry, Patras University General Hospital, Faculty of Medicine, School of Health Sciences, University of Patras, Patras, Greece; 7 Patras Dementia Day Care Centre, Corporation for Succor and Care of Elderly and Disabled–FRODIZO, Patras, Greece; 8 Department of Psychiatry and Psychotherapy, University Hospital, LMU Munich, Munich, Germany; 9 German Centre for Neurodegenerative Diseases (DZNE) Munich, Munich, Germany; 10 Munich Cluster for Systems Neurology (SyNergy), Munich, Germany; 11 Sheffield Institute for Translational Neurosciences (SITraN), University of Sheffield, Sheffield, UK; 12 Division of Mental Health of Older Adults, Department of Psychiatry and Psychotherapy, Ludwig-Maximilians-Universität München, Nußbaumstr. 7, 80336 Munich, Germany

**Keywords:** miRNA, preclinical, blood-biomarker, omics, GWAS

## Abstract

**Background:**

Identifying individuals before the onset of overt symptoms is key in the prevention of Alzheimer’s disease (AD).

**Objkectives:**

Investigate the use of miRNA as early blood-biomarker of cognitive decline in older adults.

**Design:**

Cross-sectional.

**Setting:**

Two observational cohorts (CHARIOT-PRO, Alzheimer’s Disease Neuroimaging Initiative (ADNI)).

**Participants:**

830 individuals without overt clinical symptoms from CHARIOT-PRO and 812 individuals from ADNI.

**Measurements:**

qPCR analysis of a prioritised set of 38 miRNAs in the blood of individuals from CHARIOT-PRO, followed by a brain-specific functional enrichment analysis for the significant miRNAs. In ADNI, genetic association analysis for polymorphisms within the significant miRNAs’ genes and CSF levels of phosphorylated-tau, total-tau, amyloid-β42, soluble-TREM2 and BACE1 activity using whole genome sequencing data. Post-hoc analysis using multi-omics datasets.

**Results:**

Six miRNAs (hsa-miR-128-3p, hsa-miR-144-5p, hsa-miR-146a-5p, hsa-miR-26a-5p, hsa-miR-29c-3p and hsa-miR-363-3p) were downregulated in the blood of individuals with low cognitive performance on the Repeatable Battery for the Assessment of Neuropsychological Status (RBANS). The pathway enrichment analysis indicated involvement of apoptosis and inflammation, relevant in early AD stages. Polymorphisms within genes encoding for hsa-miR-29c-3p and hsa-miR-146a-5p were associated with CSF levels of amyloid-β42, soluble-TREM2 and BACE1 activity, and 21 variants were eQTL for hippocampal *MIR29C* expression.

**Conclusions:**

six miRNAs may serve as potential blood biomarker of subclinical cognitive deficits in AD. Polymorphisms within these miRNAs suggest a possible interplay between the amyloid cascade and microglial activation at preclinical stages of AD.

**Electronic Supplementary Material:**

Supplementary material is available in the online version of this article at 10.14283/jpad.2023.99.

## Introduction

Preclinical Alzheimer’s disease (AD) is receiving increased attention as a window of opportunity for therapies aiming at slowing disease progression (1). AD pathology may start several decades before the onset of overt symptoms, accompanied by characteristic biomarker changes in the CSF (2). More recently, blood levels of p-tau181 and p-tau217 have shown promise as biomarker candidates for predicting AD dementia in cognitively unimpaired individuals (3, 4), but they remain to be validated fully.

Blood microRNAs (miRNAs) are attracting considerable interest as novel minimally invasive biomarker candidates for AD. MiRNAs can be measured in any body fluid or tissue, and we have recently reported a consistent and reproducible dysregulated expression in the CSF, brain and blood of patients with AD (5). Moreover, measurement of selected miRNAs in the blood shows good performance in differentiating healthy controls from individuals with AD (6). Recently, a signature of three miRNAs (miR-181a, miR-148a and miR-146a) was reported to correlate with cognitive function in cognitively healthy individuals. The authors also reported increased blood levels of these three miRNAs in individuals progressing from MCI to AD within two years (7).

Interestingly, few studies have investigated the role of miRNAs in preclinical stages of AD. Yet, several miRNAs are directly involved in pathways initiating early pathological changes. For instance, miR-146a, downregulated in the blood of AD patients, represses the expression of neurofilament light chains (NfL), which are reported to be increased several years before the onset of clinical symptoms in individuals carrying a mutation in the APP, *PSEN1* or *PSEN2* familial AD genes (8, 9). Similarly, miR-125b, also downregulated in AD, blocks the translation of SphK1, a mediator of neuroinflammation in early stages (10). In parallel, the development of large-scale genome wide analysis techniques has offered novel insights into the role of miRNA gene polymorphisms in the development of neurological disorders (11). Mutations within a miRNA gene may affect different stages of its transcript processing and may result in abrogated miRNA function (12). In AD, the presence of rs2910164 within *MIR146A* resulted in decreased levels of miR-146a-5p, correlating with increased levels of TLR-2, a critical microglial receptor mediating neuroinflammation following its binding to Aβ42 (13, 14). Similarly, the presence of rs6070628 within *MIR298* correlated with higher CSF levels of p-tau181 in a dose-dependent fashion (15).

The aim of this study was to investigate the expression of selected blood miRNAs in healthy subjects at different level of cognitive performance. We prioritised significantly dysregulated miRNAs in AD based on our recent systematic review and meta-analysis of the literature (5). In subsequent steps to explore the biological underpinnings of our miRNA targets, we performed pathway enrichment analyses and explored associations between SNPs within genes coding for miRNAs dysregulated in AD and common CSF markers of neurodegeneration.

## Methods

### Study populations

For the miRNA expression analyses, we accessed the biobanked samples of the prospective, observational CHARIOT PRO Main Study cohort (Cognitive Health in Ageing Register: Investigational, Observational, and Trial studies in dementia research: Prospective Readiness cOhort, clinicaltrials.gov ID NCT02114372), recruited in West London (UK) between February 2014 and December 2016. The study was terminated in 2017 and was replaced by the still on-going prospective longitudinal biomarker-enriched CHARIOT PRO Sub-Study. Subjects were aged 60 to 85 and were at different levels of cognitive performance based on their Repeatable Battery for the Assessment of Neuropsychological Status (RBANS) score at baseline. Subjects with a diagnosis of AD or MCI based on the criteria of MCI or AD dementia per the 2011 National Institute on Aging-Alzheimer’s Association (NIA-AA) recommendations were excluded (16, 17). Furthermore, during the screening process, a multidisciplinary adjudication panel (including neurologists, psychiatrists, and neuropsychologists) evaluated individuals with any baseline RBANS index below 1.5 SD. Participants whose low performance score were likely to be attributable to undiagnosed cognitive impairment were excluded from the study. Further exclusion criteria included presence of any neurological or psychiatric condition, substance use disorder or the presence of reversible causes of dementia. Further details can be found elsewhere (18).

For the genetic association analysis with CSF biomarkers, we used data obtained from the Alzheimer’s Disease Neuroimaging Initiative (ADNI) database (adni.loni.usc.edu). The ADNI was launched in 2003 as a public-private partnership, led by Principal Investigator Michael W. Weiner, MD. The primary goal of ADNI has been to test whether serial magnetic resonance imaging (MRI), positron emission tomography (PET), other biological markers, and clinical and neuropsychological assessment can be combined to measure the progression of mild cognitive impairment (MCI) and early Alzheimer’s disease (AD).The ADNI cohort includes subjects with AD dementia, MCI and healthy controls (19). AD dementia was diagnosed based on the National Institute of Neurological and Communicative Disorders and Stroke-AD and Related Disorders Association (NINCDS-ADRDA) criteria. Individuals with AD had a Mini-Mental State Examination (MMSE) score of 20–26 and a Clinical Dementia Rating (CDR) score of 0.5 or 1. Individuals without functional complaints but a MMSE score of 24–30, a CDR score of 0.5 (with a memory box score of 0.5 or greater) and memory complaints were classified as MCI. Healthy controls were free of memory complaints, impairment on cognitive testing and were independent in their activities of daily living (19). For both cohorts, subjects provided written informed consent.

### Neuropsychological measurements in CHARIOT PRO

The RBANS score was used to measure cognitive function in the CHARIOT PRO cohort. Briefly, this method assesses five cognitive domains (immediate memory, visuospatial/ constructional, language, attention and delayed memory) using 12 tests. The individual five tested domains are summed in a total score, subsequently adjusted to a reference dataset, including 540 healthy subjects 20–89 years old (20). The RBANS was validated in community dwelling individuals and is used as a screening tool for dementia in clinical practice and trials (21). In a previous study, 1.5 standard deviation (SD) below the mean performed best when differentiating MCI patients from healthy controls, while 2 SD below the mean identified individuals with more advanced stages and functional impairment. Furthermore, the lower end of normal performance was identified as 1 SD below the mean (22). Therefore, we defined subtle cognitive deficits in overall cognitively normal individuals using a cut-off of 1 SD below the mean total RBANS score.

### qPCR analysis in CHARIOT PRO

In total, 46 miRNAs were analysed, including 38 prioritised candidate miRNAs (32 in the blood and six in the brain) (5) selected based on our recent systematic review and meta-analysis of over 100 studies and another eight endogenous miRNAs with reportedly stable expression in the blood (23). Two spike-in controls, UniSp3 and UniSp6, were used as serial quality controls throughout the qPCR process. Blood samples were collected in PAXgene Blood RNA tubes (Qiagen, Venlo, The Netherlands). Subsequent qPCR analyses were performed on the miRCURY LNA miRNA PCR System at Qiagen laboratories. Further details can be found in the supplementary material.

During quality control of the qPCR data, qPCR cycle threshold (Ct) values > 35 (i.e., the number of amplification cycles needed for the target to be detected above the background signal) were considered not available, according to the manufacturer’s recommendations. MiRNAs with Ct values > 35 in more than 50% of the samples were removed (total miRNAs removed: n=18 (39.13%), which is comparable to other studies (24)) (eTable 1 in the Supplement). Finally, normalization was undertaken using the geNorm algorithm, preferred over other normalization approaches according to a recent comparative study (24). In a final step, Ct values were converted to fold changes.

### Whole genome sequencing in ADNI

Whole genome sequencing (WGS) data were provided by the ADNI genetics core team. The analyses were undertaken using the Illumina HiSeq 2000 platform and followed the Genome Analysis Toolkit (GATK) pipeline. Following download of the ADNI data in PLINK format, we conducted additional quality control procedures based on a previously described protocol (25). In brief, after a check of discordant sex information, we removed individuals with high missing and outlying heterozygosity rates (cut-off call rate > 0.03, cut-off heterozygosity > 3 SD), duplicated and related individuals (cut-off Pi-hat 0.2). Genotyping imputation was performed using the TOPMed Imputation server, shown previously to improve the imputation accuracy for rare variants (26); variants with poor imputation quality r2 < 0.3 were excluded.

### MiRNA gene region definition

The region of interest was defined before and after the start of the miRNA gene region based on the GRCh38 genome assembly. Haplotype blocks were identified using the Gabriel algorithm implemented in Haploview. This method uses D prime, a marker of linkage disequilibrium (LD), between two SNPs to identify a haplotype block defined as the presence of 95% SNPs in strong LD. A pair of SNPs are in strong LD having the one-sided upper 95% confidence bound on D′ is >0.98 (consistent with no historical recombination) and the lower bound D′>0.7.

### Biomarker measurements in ADNI

CSF biomarker measurements and associated data were downloaded from ADNI. All analyses followed ADNI standard operating procedures and published protocols. Briefly, amyloid beta 42 (Aβ342), total-tau (t-tau) and phosphorylated tau 181 (p-tau181) analyses were performed on the multiplex xMAP platform (Luminex Corp., Austin, TX, USA) with monoclonal antibodies specific for Aβ42 (4D7A3), t-tau (AT120), and p-tau181 (AT270) (27). Soluble triggering receptor expressed on myeloid cells 2 (sTREM2) was measured using a previously described ELISA protocol (28). Finally, beta-site amyloid precursor protein cleaving enzyme 1 (BACE1) activity levels in the CSF were measured using a two-step assay described previously (29).

### ATN classification in ADNI

We used the ATN classification from the National Institute on Aging-Alzheimer’s Association research framework to identify individuals with Alzheimer’s Disease Related Dementia (ADRD). As described before, A+ was defined as CSF Aβ42 ≤ 192 pg/mL while TN+ was defined as p-tau181 ≥ 23 pg/mL and/ or t-tau ≥ 93 pg/mL (30).

### Statistical analysis

Distribution of continuous variables was assessed for normality using the Kolmogorov Smirnov test and log10 transformed when appropriate. Demographic differences between the RBANS low performance and normal performance groups were evaluated with a Student’s t-test. Mann-Whitney test was used for non-normally distributed variables and Pearson’s Chi Square test for categorical variables.

For the qPCR analysis, we used partial correlation (Pearson correlation for normally distributed values and Spearman rank correlation for non-normally distributed values) adjusted for age and sex to investigate the relationship between miRNA concentration and RBANS score. Subsequently, a multivariate regression adjusted for age, sex, education status (<13 education years, ≥13 education years), ethnicity and apolipoprotein E (*APOE*) ε4 carrier status (defined as the presence of at least one e4 allele) was applied, with miRNA Ct values as dependent variable. Using the same covariates, we assessed differences in miRNA concentrations between the two RBANS performance groups using ANCOVA. Finally, for the miRNAs showing a significant difference between the two performance groups we constructed a ROC (receiver operating characteristic) curve and calculated their AUC (area under the curve) to investigate the discrimination ability of the miRNAs.

Genetic association analysis for CSF biomarkers was undertaken in PLINK v1.07 using linear regression considering an additive genetic model. The following covariates were added in the model: SNP, age, sex, *APOE*ε4-carrier status and diagnosis at baseline. We controlled for population structure by adjusting for the first five principal components. Finally, we conducted a Pearson correlation and a multivariate regression analysis (including the above-mentioned covariates) between biomarkers significantly associated with polymorphisms of the same miRNA gene.

Confidence intervals of 95% were considered. For the miRNA expression analysis, we applied a Benjamini Hochberg FDR adjustment to account for multiple testing. This approach was also selected for the SNP association analysis as several analysed SNPs were in LD and not independent from each other. Statistical significance was set at FDR α<0.05. In a final step we conducted a subgroup analysis focusing only on individuals with normal cognition (labelled “CN” in ADNI) and belonging to the following ATN groups: A+TN+, A+TN− and A−TN+.

All analyses were undertaken in R v4.1.2. Baseline characteristics are described as mean ± SD.

### Post-hoc analyses

For the significant miRNAs, we conducted a pathway enrichment analysis following a previously described protocol using EnrichmentMap and g:Profiler (31). First, we extracted experimentally validated miRNA gene pairs from miRTarbase and to be more AD-specific, selected only genes highly expressed in the brain according to the Human Protein Atlas (HPA) (32). Further details on the tissue specificity methodology is described elsewhere (32). Then, we conducted a functional enrichment in Gene Ontology, KEGG, REACTOME, considering only gene sets with a minimum of 5 and maximum of 500 genes. Background genes consisted of all genes highly expressed in the brain. The results were then visualised using the plugin Enrichment Map in Cytoscape to explore an overlap in significantly enriched gene sets between different miRNAs. The Jaccard and overlap coefficient thresholds were set to 0.25, a conservative threshold to identify overlapping terms. Finally, pathways were grouped manually into families of biological function. Overlap between miRNAs gene targets were visualised using the UpSetR package for R. For SNPs significantly associated with CSF biomarkers of AD, we undertook a series of post-hoc functional analyses while focusing on hippocampus cell lines. First, co-localisation of SNPs with expression quantitative trait loci (eQTL) signals were identified using BRAINEAC (The brain eQTL Almanac), providing eQTL data for 10 brain regions obtained from 134 healthy individuals. Second, we used HaploReg v4.1 to obtain regulatory information from the Roadmap Epigenomics project for histone modification marks, chromatin states and DNase hypersensitivity. The effects on regulatory motifs (also called transcription factor (TF) binding sites) were obtained from Chromatin Immunoprecipitation (ChIP) sequencing data from the ENCODE project. Finally, risk SNPs for disease were obtained from VARAdb v1.0 which uses a data mining approach over NHGRI GWAS catalogue, GWASdb v2.0, GAD, and GRASP. A detailed view of the bioinformatics tools used can be found in the supplementary material (eTable 2 in the Supplement)

## Results

### Characteristics of the CHARIOT PRO and ADNI cohorts

The CHARIOT PRO cohort included 830 individuals with mean age of 68.70 ± 3.51 years and including 475 (57.23%) female participants (Table 1). The 1SD below the mean cut-off for subtle cognitive deficits (1 SD below the mean) corresponded to an RBANS Total Scale score of 88, and 132 participants (15.90%) fell at or below this level. The two performance groups showed a statistically significant difference in sex (P=0.007), and education status (P=0.008) (Table 1). The ADNI cohort for whom WGS data was available consisted of 812 individuals. After quality control, 62 individuals were removed (including individuals with non-European ancestry). The total number of markers with minor allele frequency (MAF) >0.05 and call rate > 0.9 was 6 209 511. No markers deviated from the Hardy Weinberg Equilibrium at a P < 10–6. The sample’s mean age was 73.42 ± 7.03 years, and 322 (42.93%) participants were female (Table 1). For this ADNI sub-cohort, 580, 573, 580, 187, and 114 individuals had data available for Aβ42, t-tau, p-tau181, sTREM2, BACE1 CSF measurements, respectively (Table 1).

**Table 1:** Demographics CHARIOT PRO and ADNI cohorts

**CHARIOT-PRO**						
			**ALL**	**Low performance group**	**Normal performance group**	**P value**
N			830	132	698	
Age (years)			68.70 ± 3.51	69.22 ± 4.83	68.68 ± 3.45	*
Female: n (%)			475 (57.23)	61 (46.21)	414 (59.40)	**
Male: n (%)			355 (42.77)	71 (53.79)	283 (40.60)	
*APOEε*4 carrier			168 (20.24)	34 (25.76)	134 (19.20)	
Education ≥ 13 years n (%)			431 (51.93)	54 (40.91)	377 (54.01)	**
Race						***
White: n (%)			736 (88.7%)	96 (72.7%)	640 (91.7%)	
Black or African American: n (%)			6 (0.7%)	5 (3.8%)	1 (0.1%)	
Asian: n (%)			36 (4.4%)	20 (15.2%)	16 (2.3%)	
Multiple: n (%)			10 (1.2%)	5 (3.8%)	5 (0.7%)	
Other: n (%)			12 (1.4%)	4 (3%)	8 (1.2%)	
Unknown: n (%)			30 (3.6%)	2 (1.5%)	28 (4%)	
RBANS						
immediate			103.21 ± 14.74	83.41 ± 12.89	106.96 ± 11.78	***
			[94.00–112.00]	[75.25–90.00]	[100.00–114.00]	
			(49.00–144.00)	(49.00–123.00)	(69.00–144.00)	
constructional			97.87± 16.09	82.11 ± 14.19	100.85 ± 14.63	***
			[87.00–109.00]	[72.00–92.00]	[92.00–112.00]	
			(50.00–131.00)	(50.00–121.00)	(58.00–131.00)	
language			104.21 ± 13.38	89.23 ± 12.77	107.05 ± 11.48	***
			[96.00–112.00]	[83.00–98.00]	[99.00–116.00]	
			(51.00–134.00)	(51.00–116.00)	(79.00–134.00)	
attention			104.44 ± 16.05	87.26 ± 12.30	107.69 ± 14.54	***
			[94.00–115.00]	[79.00–94.00]	[97.00–118.00]	
			(49.00–146.00)	(49.00–132.00)	(68.00–146.00)	
delayed memory			100.41 ± 11.93	86.67 ± 12.43	103.01 ± 9.89	***
			[95.00–107.00]	[80.25–95.00]	[98.00–110.00]	
			(40.00–134.00)	(40.00–113.00)	(56.00–134.00)	
TOTAL			102.72 ± 13.83	80.96 ± 6.61	106.85 ± 10.60	***
			[94.00–112.00]	[78.00–86.00]	[99.00–114.00]	
			(55.00–143.00)	(55.00–88.00)	(89.00–143.00)	
ADNI						
	**ALL**	**Aβ42**	**t-tau**	**p-tau181**	**sTREM2**	**BACE1 activity levels**
N	750	580	573	580	187	114
Age (years)	73.42 ± 7.03	73.03 ± 7.12	73.04 ± 7.14	73.03 ± 7.12	72.85 ± 6.82	74.45 ± 6.23
Female: n (%)	322 (42.93):	250 (43.1):	248 (43.28):	250 (43.1):	71 (37.97):	41 (35.96):
Male: n (%)	428 (57.07)	330 (56.9)	325 (56.72)	330 (56.9)	116 (62.03)	73(64.04)
*APOEε*4 carrier	306 (40.80):	231 (39.83):	227 (39.62):	231 (39.83):	75 (40.11):	43 (37.72):
	444 (59.20)	349 (60.17)	346 (60.38)	349 (60.17)	112 (59.89)	71 (62.28)
Education ≥ 13 years: n (%)	638 (85.07)	497 (85.69)	491 (84.66)	497 (85.69)	157 (83.51)	97 (85.09)
Diagnosis (CN/MCI/ AD)	255/450/45	188/350/42	187/347/39	188/350/42	47/140/0	55/59/0
Biomarkers		179.75 ± 54.01	83.15 ± 45.82	38.69 ± 23.02	4599.19 ± 2576.53	49.08 ± 20.04

Legend: P values: * α <0.05, ** α < 0.01, *** α < 0.001; RBANS values are presented as mean ± SD [IQR] (range), other variables are presented as mean ± SD

### miRNA and cognitive performance in CHARIOT PRO

**Figure 1:**
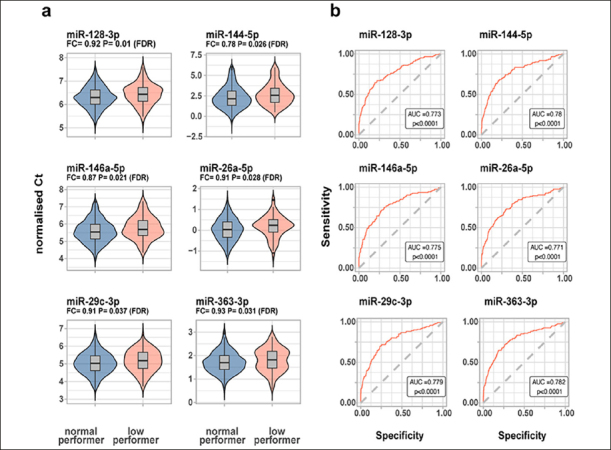
MiRNA expression levels in healthy individuals in CHARIOT PRO (n=830) a) miRNAs significantly dysregulated between the normal vs low performance group, with higher Ct values corresponding to lower expression b) ROC curves for significant miRNAs. Legend: In figure B, the ROC p value reflects the significance level of the deviation from the null hypothesis (i.e. AUC = 0.50)

In the unadjusted analysis, 17 miRNAs were correlated with the RBANS index scores or the total scale. In a multivariate regression analysis, adjusted for age, sex, education years, ethnicity and *APOE*ε4 carrier status, the RBANS language index was significantly (FDR α = 0.05) associated with hsa-let-7a-5p, hsa-let-7c-5p, hsa-let-7d-5p, hsa-miR-144-5p, hsa-miR-93-5p and hsa-miR-98-5p. Furthermore, we found hsa-miR-363-3p to be significantly (FDR α = 0.05) associated with the RBANS attention index. Finally, hsa-miR-144-5p was significantly (FDR α = 0.05) associated with the RBANS total scale (eTable 3 in the Supplement). When comparing miRNA expression levels between the two RBANS performance groups, six miRNAs (hsa-miR-128-3p, hsa-miR-144-5p, hsa-miR-146a-5p, hsa-miR-26a-5p, hsa-miR-29c-3p, hsa-miR-363-3p) were downregulated in the low performance group compared to the normal performance group (Figure 1). A significant direct correlation (ranging from rs = 0.09 to rs = 0.55) was found between the six miRNAs (eTable 4 and eFigure 1 in the Supplement). It is noteworthy that the six miRNAs had similar diagnostic performance, with the hsa-miR-363-3p ROC curve having the highest AUC [95% CI] = 0.78 [0.74–0.83] (Figure 1). Combining the miRNA did not improve the AUC (AUC [95% CI] = 0.78 [0.73–0.83]), which may be explained by the strong correlation between the six miRNAs.

### Brain specific pathways enrichment analysis for the significant miRNAs

**Figure 2:**
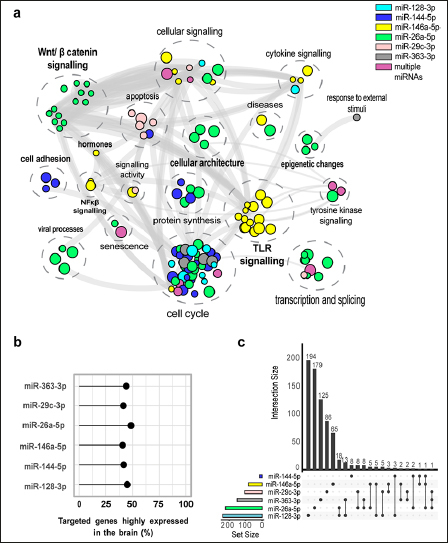
Functional enrichment for associated SNPs a) Pathway enrichment analysis for the significant miRNAs differentially expressed between low and normal performers in CHARIOT PRO. In bold are clusters for which pathways are enriched by genes targeted by a single dysregulated miRNA. b) Percentage of targeted brain specific genes for the significant miRNAs c) Intersection between targeted brain specific genes for each significant miRNA.

For the six miRNAs significantly dysregulated in the low performance group, we identified 1 641 experimentally validated miRNA-gene pairs in miRTarBase. Of these, 734 (44.71%) were highly expressed in the brain. MiR-128-3p targets the highest number of genes while hsa-miR-26a-5p targets the highest percentage of genes highly expressed in the brain (48.58%) (Figure 2). Although there was no overlap between the targeted genes for the six miRNAs, two genes (*MDM2, SMAD4*) were targeted by at least three of the dysregulated miRNAs (Figure 2). The pathway enrichment analysis conducted in KEGG, GO and REACTOME for the brain specific miRNA-gene pairs revealed 152 unique pathways (Figure 2). Importantly, nine pathways were targeted by more than one miRNA; these pathways contribute to cell cycle regulation, transcription and splicing, cellular signalling and tyrosine kinase signalling. Moreover, the pathway cluster “Wnt/ β catenin signalling” was targeted by hsa-miR-26a-5p only, while pathways clusters related to inflammation such as “NF-κβ signalling”, “cytokine production” and “Toll-like receptor signallingβ contained pathways were targeted by hsa-miR-146a-5p only (eTable 5 in the Supplement).

### Polymorphisms within the significant miRNA genes and their association with CSF biomarkers

**Figure 3:**
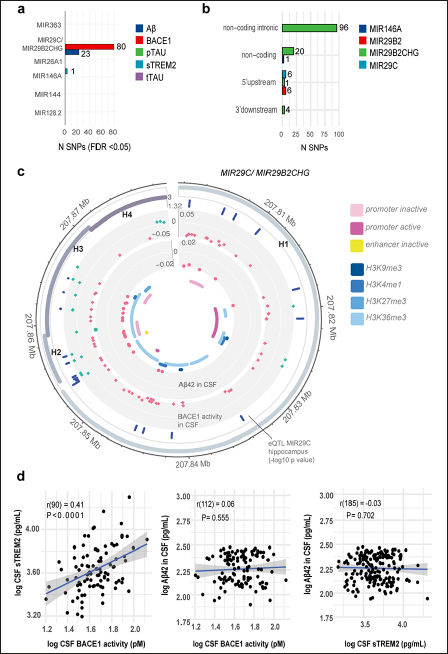
Genetic association results and post-hoc analysis for polymorphisms within the significant miRNA genes a) Number of SNPs significantly associated (FDR <0.05) with biomarkers of neurodegeneration (by miRNA gene). b) Functional consequence of the significant SNPs. c) Circos plot showing the genetic association analysis results for the significant SNPs located within the *MIR29B2CHG* gene including haplotype blocks (H1–H4), SNPs which were significant eQTL for MIR29C expression in the hippocampus (in BRAINEAC), estimate (β) from the association analysis for Aβ and BACE1 activity levels, histone modification marks in hippocampal cells. d) Correlation plots between CSF biomarkers for which SNPs located within *MIR29B2CHG* and *MIR146A* share common genetic association.

After identifying the six dysregulated miRNAs in individuals with lower cognitive performance, we utilised ADNI data to investigate whether polymorphisms within these miRNA genes are associated with altered levels of neurodegeneration biomarkers. The six miRNA genes were located on six different chromosomes and their lengths varied from 76b for *MIR26A1* to 77.6 kb for MIR29B2CHG, the host gene of miR-29c-3p. Most of the variants at MAF > 0.05 were located within *MIR29B2CHG* (196 variants); one variant was found in *MIR146A* (Figure 3). The SNPs fell into different effect categories, with the majority being non-coding intronic. Six SNPs were located in the 5′ upstream region of *MIR29B2CHG* (Figure 3). After adjusting for multiple comparison, our association analysis found that 80 variants within *MIR29B2CHG* were significantly associated with BACE1 activity in the CSF (PFDR<0.05). Of these, 23 variants were also significantly associated with Aβ42 CSF levels. The variants showed mixed effects on Aβ and BACE1 activity levels: all SNPs except one were significantly associated with decreased Aβ CSF levels, 20 and 40 SNPS were associated with increased and decreased BACE1 activity levels, respectively (Figure 3). Finally, minor allele carriers for rs2910164, the variant located in *MIR146A*, showed decreased sTREM2 CSF levels (β=−0.063 95%CI [−0.123; −0.002], p=0.043).

Considering that polymorphisms within *MIR29B2CHG* and *MIR146A* affected the expression of CSF levels of Aβ42, BACE1 activity and sTREM2, we explored the relationship between these biomarkers. We undertook a multivariate regression analysis and found a positive correlation between log BACE1 activity (r=0.41, p<0.0001) and log sTREM2 levels in the CSF (β=0.34, 95% CI [0.18,0.49]): for a 10% increase in BACE1 activity in the CSF; the sTREM2 levels in the CSF increased by 3.3% (Figure 3).

### Subgroup analysis

The subgroup analysis including only cognitive normal individuals with elevated ADRD biomarkers yielded significant associations between polymorphisms located in five miRNA genes and CSF levels of p-tau181, t-tau, BACE1 activity and sTREM2. However, no variants remained significant after adjustment for multiple comparison (eTable 6).

### Post hoc analysis of the significant polymorphisms

A closer inspection of the significant variants showed that 21 variants acted as significant eQTL for hsa-miR-29c-3p expression in the hippocampus in BRAINEAC. Also, data from the Roadmap Epigenomics project revealed that these SNPs are in regulatory regions with enhancer/promoter activity in hippocampal cells. Noteworthy, according to ChIP data from the ENCODE project, these SNPs altered the binding of several brain specific transcription factors, critical in neurogenesis, apoptosis or neuronal response to Aβ disposition (Table 2, eTable 7 in the Supplement).

**Table 2:** Significant SNPs in MIR29B2CHG with eQTL for MIR29C expression in the hippocampus

**rsID**	**chr**	**Pos hg38**	**ref**	**alt**	**predicted function**	**motifs**	**phenotype**
rs2910164	5	160485411	C	G	non-coding		NA
rs56075814	1	207802604	T	C	5′ upstream	SIX5_disc4	glioma
rs11118612	1	207806232	T	A	non-coding	BATF_disc3; Ets_known3; Mrg_2; SP2_disc2; STAT_known4; Tgif1_2	glioma
rs4844620	1	207807556	G	A	non-coding	Pax-8_1; SREBP_known3	glioma
rs66532523	1	207810400	A	C	non-coding	ELF1_known1; Egr-1_disc4; Ets_known9; Msx-1_1; STAT_known4	glioma
rs4844392	1	207817864	C	G	non-coding intronic	HNF4_disc3; Mtf1_1	chronic central serous retinopathy, white blood cell count
rs4844393	1	207821084	T	C	non-coding intronic		glioma
rs61821293	1	207833932	T	G	non-coding intronic	Evi-1_2; **GATA_known1**	glioma
rs1318653	1	207841577	T	C	non-coding intronic	**Foxj2_2**	age-related macular degeneration, febrile seizures
rs11118668	1	207844570	C	T	non-coding intronic		glioma
rs55935450	1	207845434	T	A	non-coding intronic	HNF4_disc5; Maf_known1; Nrf-2_2	glioma
rs7532674	1	207853394	G	T	non-coding intronic	CCNT2_disc2; NRSF_known3; **SP1_disc3; SP2_disc3**	NA
rs926631	1	207853632	T	C	non-coding intronic	EBF_disc1; EBF_disc2; EBF_known1; EBF_known2; EBF_known3; GR_disc4; NF-kappaB_disc2; YY1_disc1; YY1_known4	infant head circumference, obesity with early age of onset
rs2745981	1	207853847	C	T	non-coding intronic		refractive error, obesity with early age of onset
rs2745982	1	207854102	A	C	non-coding intronic	PRDM1_known1; Pou2f2_known8; Pou3f3	glioma
rs2796241	1	207854114	C	A	non-coding intronic	Pou2f2_known8; Pou3f3	urinary metabolites
rs2796242	1	207855716	G	A	non-coding intronic	BCL_disc8; ETF; HEN1_1; HEY1_disc2; Myf_3; Rad21_disc7; SMC3_disc3	glioma
rs2796244	1	207856494	G	A	non-coding intronic		glioma
rs984984	1	207861743	G	T	non-coding intronic		obesity with early age of onset
rs1319388	1	207861794	C	A	non-coding intronic	**YY1_known6**	glioma
rs2796247	1	207863631	C	T	non-coding intronic	**RXRA_known4**	primary rhegmatogenous retinal detachment, obesity with early age of onset (age >2)
rs2724362	1	207865251	G	T	non-coding intronic	HNF1_7; Lhx3_2; STAT_known1; STAT_known2; Zfp187	glioma

Legend : in bold, transcription factors with role in neurogenesis or AD specific biological pathways (details can be found in supplementary table 6)

## Discussion

In this study, we dissected miRNA blood expression levels in 830 older individuals without MCI or dementia. We studied 38 miRNAs significantly associated with AD in our recent systematic literature review and meta-analysis (5). Six miRNAs (hsa-miR-128-3p, hsa-miR-144-5p, hsa-miR-146a-5p, hsa-miR-26a-5p, hsa-miR-29c-3p and hsa-miR-363-3p) were significantly downregulated in the blood of individuals with subtle cognitive deficit on the RBANS. This set of miRNAs could serve as predictive biomarker in individuals with pre-MCI cognitive deficits, since they may be dysregulated before the onset of overt clinical AD. To unveil the mechanistic role of the six-miRNAs signature, we undertook a genetic association analysis using 750 individuals from ADNI. Here, we found that SNPs within the genes coding for miR-29c-3p and miR-146a-5p were significantly associated with Aβ442, BACE1 activity and sTREM2 levels in the CSF. Our pathway enrichment analysis suggests a role for miR-29c in early phases of the disease. Supporting our results, a recent study in 76 subjects with MCI found that miR-146a-5p belonged to a set of three blood miRNAs (miR-181a-5p, miR-148a-3p and miR-146a-5p) predicting progression to AD dementia (7). The authors suggested that targeting this miRNA signature via RNA therapeutics ameliorates cognitive decline in an AD mouse model (7). MiR-181a-5p and miR-148a-3p were not associated with AD in our previously conducted meta-analysis (5). However, the authors identified the target miRNAs in a cohort of healthy younger individuals; miR-181a-5p and miR-148a-3p may be differentially expressed several decades before disease onset, but normally expressed closer to the first symptoms (7).

Our in-silico pathway enrichment analysis showed that the set of six circulating miRNAs targets brain-specific genes involved in early AD pathological processes, including apoptosis and TLR and NF-κβ inflammatory signalling pathways. Strikingly, we found that genes from the Wnt/β catenin system are targeted exclusively by miR-26a. Mounting evidence shows that this pathway plays a key role in AD: decreased levels of Wnt/β catenin were associated with increased Aβ deposits and impaired cognitive function in an AD mouse model (33). Moreover, β catenin also inhibited BACE1 expression leading to decreased Aβ formation (34). So far, the relationship between miR-26a and the Wnt/β catenin system has not been explored in AD. In non-small cell lung cancer and cholangiocarcinoma, downregulation of miR-26a lead to lower β catenin levels (35). Consequently, we could hypothesize that individuals in our cohort with lower cognitive performance and downregulated miR-26a had lower levels of Wnt and β catenin, resulting in decreased Aβ clearance. This hypothesis is of particular interest, since Wnt/ β catenin is targeted by statins, a class of drugs possibly reducing AD risk (36). In addition to miR-26a, we found that miR-146a targets brain specific genes involved in the NF-κβ and TLR signalling pathways. These two pathways are key actors in early AD pathological processes (37). For instance, NF stimulates the expression of pro- and anti-inflammatory cytokines, such as IL1-β and TNF-α, before Aβ deposition (38).

So far, no other study has investigated the expression of miR-29c in a longitudinal cohort of patients from the AD continuum. Our pathway enrichment analysis suggests a role for miR-29c in early phases of the disease. Notably, we showed how miR-29c targets brain specific genes of the caspase family, playing a role early in AD (39). In line with our results, a recent study showed that administration of a caspase-1 inhibitor to pre-symptomatic APP mutant mice slowed down cognitive decline (40). Moreover, in a Parkinson’s disease mouse model, decreased intraneuronal a synuclein aggregation was reported following miR-29c induced inhibition of pro-inflammatory cytokines production and caspase-3 and caspase-9 expression (41). Therefore, early downregulation of miR-29c at preclinical stages of AD may promote the development of Aβ deposits by increasing the production of pro-inflammatory cytokines.

We identified a positive correlation between BACE1 activity and sTREM2 levels in the CSF, suggesting an interplay between microglial activation and the amyloid cascade at early stages of the disease. To the best of our knowledge, this relationship has not been described so far in the literature. In individuals with MCI, both increased BACE1 activity and increased microglial activation have been reported on brain extracts and 11C-®PK11195 PET imaging (42, 43). Moreover, in an AD mouse model, early disease stages were characterised by microglial activation with increased expression of IL-1β and IL-6 cytokines as well as increased BACE1 activity (44). Nevertheless, the relationship between BACE1 and microglial activation needs to be explored further as one study reported an inverse relationship between sTREM2 and BACE1 activity: in an APP transgenic mouse, inhibiting BACE1 expression resulted in an increase in microglial activation (45). Our results suggest a complex interplay between BACE1 and microglial activation, and different stages of microglial activation within participants of the BACE1 inhibitor trials might have undermined the efficacy of the substances, contributing to the disappointing results (46).

We found that 21 variants significantly associated with Aβ and BACE1 CSF levels were eQTL for miR-29c-3p expression in the hippocampus of neuropathologically normal individuals. These findings shed a further light on the early interplay between miR-29c-3p and biological pathways involved in AD pathogenesis and open the discussion on the potential of miR-29c-3p as novel therapeutic candidate. Interestingly, miR-29c-3p and two other dysregulated miRNAs target MDM2, an important regulator of the p53 signaling pathway and whose inhibition has gained increasing attention in the treatment of cancers and auto-immune disorders (47). Moreover, a recent study showed that MDMD2 inhibition in mice improved neurogenic deficits in an experimental mice model of fragile X syndrome (48). Future studies are needed to explore the potential of miR-29c mimic as an MDM2 inhibitor.

In this study, we defined individuals with subtle cognitive deficits using a −1 SD cut-off on the RBANS total scale, based on previous reports from the literature for MCI and AD patients (21, 22). This cut-off may result in cognitive normal adults with lower education/ premorbid functioning being misclassified as low cognitive performer. Nonetheless, when comparing our low performance group’s RBANS total scores with other papers, we observed that the scores were close to scores obtained by MCI participants. Hammers et al reported a RBANS total score of mean (SD) 81.6 (11.3) for clinic amnestic MCI participants vs 109.2 (12.7) for cognitively intact community dwelling older adults (49). Similarly, in a cohort of 162 individuals from a medical center, MCI participants scored a RBANS total score of mean (SD) 81.8 (11.1) vs 103.4 (14.2) for healthy controls (21).

Some limitations of our study need to be mentioned. First, the population-based CHARIOT PRO cohort does not include neurodegenerative biomarker data and no long-term follow-up investigations. To offset those limitations, we used CSF results from the memory clinic-based ADNI cohort, including deep longitudinal clinical and biological phenotyping. Second, we were underpowered to investigate associations for less common SNPs, such as those located within the miRNA genes. Third, we were not able to replicate our miRNA expression findings in ADNI as no such data was available. Finally, the biological plausibility of circulating miRNAs associated with disease should be underpinned ideally by similar results for their expression in brain. Since neither brain data was available for any of our cohorts nor CSF gene expression, we performed a pathway enrichment analysis based on genes known to be highly expressed in the brain, including only evidence from experimental studies, and functional analysis of the significant SNPs only conducted for findings from hippocampal cells. Investigating dysregulation of a miRNA in the brain and the blood of the same patient is limited by methodological and ethical barriers.

The major strength of our study is the careful combination of an evidence-based principled selection of the best miRNA candidates, followed by a validation in one large independent cohort, providing information for different levels of cognitive deficits and on different levels of investigation (cognitive and biological). The development of a miRNA-based point of care diagnostic solution is urgently needed for the effective roll-out of upcoming disease modifying AD therapies.

In conclusion, we provide initial experimental evidence on the diagnostic utility of a reproducible six-miRNAs signature associated with AD in a population of older individuals with subtle cognitive deficits, and provide mechanistic validation using CSF AD biomarkers. Our findings advocate the role of miRNAs as minimally invasive biomarker candidates for cognitive decline. Considering that the expression of the individual miRNAs was highly correlated, a single miRNA, such as miR-29c, could be used to flag individuals at risk for cognitive decline, followed by more detailed diagnostic assessments. This staged approach would result in a scalable, cost-effective approach with reduced burden on the affected individuals, healthcare systems and payers. Future studies on miRNA expression levels are needed to confirm our results.

### Electronic supplementary material

Supplemental Material: Association of blood microRNA expression and polymorphisms with cognitive and biomarker changes in older adults

## Data Availability

*Data sharing:* ADNI data used for this study are available from ADNI (https://adni.loni.usc.edu/data-samples/access-data/).
