# Supplementary material for: Association of Blood MicroRNA Expression and Polymorphisms with Cognitive and Biomarker Changes in Older Adults
Source: J Prev Alzheimers Dis. 2023 Sep 6;11(1):230–40. doi: 10.14283/jpad.2023.99 (PMC10994991; doi:10.14283/jpad.2023.99)
Supplement: Supplementary file 1 — Supplemental Material: Association of blood microRNA expression and polymorphisms with cognitive and biomarker changes in older adults [file 42414_2023_99_MOESM1_ESM.docx]

**Supplemental Material**

**Association of blood microRNA expression and polymorphisms with cognitive and biomarker changes in older adults.**

Sadlon Angélique MD, Takousis Petros PhD, Evangelou Evangelos PhD, Prokopenko Inga PhD, Alexopoulos Panagiotis MD, Udeh-Momoh Chinedu T PhD, Price Geraint ClinPsyD, Middleton Lefkos MD, Perneczky Robert MD for the Alzheimer’s Disease Neuroimaging Initiative

**Description of data:**

- Detailed description of qPCR analysis
- eTable 1: Percentage of NA (ie Ct values > 35) per marker; those in bold font were not included in the subsequent analyses
- eTable 2: detailed view of bioinformatics tools used
- eTable 3: Regression coefficient with RBANS as outcome variable and miRNA normalised Ct value as predictor, model adjusted for age, gender, education years, ethnicity, APOE ε4 carrier status
- eTable 4: Correlation matrix for the six significant miRNAs
- eFigure 1: Correlation matrix for the significant miRNAs
- eTable 5: Pathway enrichment analysis of the six significantly dysregulated miRNAs in the blood for targeted genes highly expressed in the brain
- eTable 6: Results from the miRNA gene association analysis including only cognitive normal individuals with positive ADRD CSF biomarkers.
- eTable 7: Role of selected brain specific transcription factors for which binding is affected by significant SNPs associated with Aβ42, BACE1 and sTREM2 levels in the CSF

**Detailed description of qPCR analysis in CHARIOT-PRO**

Briefly, RNA extraction was undertaken using the QIAsymphony PAXgene Blood RNA Kit on the QIAsymphony SP with 72 samples, in 3 batches of 24, per run, followed by spectrophotometry (Nanodrop) and gel densitometry (Agilent, TapeStation) for RNA quantification and quality control, respectively. Reverse transcription of 10 ng RNA per sample, in 10 μL reactions, was performed using the miRCURY LNA RT Kit (QIAGEN). cDNA was diluted 100x and assayed in 10 μL PCR reactions with the miRNA Ready-to-Use PCR Custom panel, using miRCURY LNA SYBR Green master mix, according to the miRCURY LNA miRNA PCR protocol; reactions, in 384-well plate format, were performed in a LightCycler 480 Real-Time PCR system (Roche), the amplification curves were visualised using the Roche LC software, Ct values determined, and provided to us for subsequent analyses.

**eTable 1: Percentage of NA (ie Ct values > 35) per marker; those in bold font were not included in the subsequent analyses**

| **miRNA** | **percentage NA (%)** |
| --- | --- |
| hsa-let-7a-5p | 3.75 |
| hsa-let-7c-5p | 0 |
| hsa-let-7d-3p | 0.94 |
| hsa-let-7d-5p | 0 |
| hsa-miR-107 | 0 |
| hsa-miR-125b-5p | 0.10 |
| hsa-miR-128-3p | 5.11 |
| hsa-miR-129-5p | 100 |
| hsa-miR-138-5p | 99.90 |
| hsa-miR-143-3p | 92.39 |
| hsa-miR-144-5p | 0.73 |
| hsa-miR-146a-5p | 1.77 |
| hsa-miR-150-3p | 92.28 |
| hsa-miR-15a-3p | 99.17 |
| hsa-miR-16-5p | 0 |
| hsa-miR-17-3p | 93.01 |
| hsa-miR-181c-5p | 98.64 |
| hsa-miR-191-5p | 0.21 |
| hsa-miR-195-5p | 99.48 |
| hsa-miR-19a-3p | 0.10 |
| hsa-miR-19a-5p | 99.79 |
| hsa-miR-21-3p | 94.99 |
| hsa-miR-210-3p | 3.34 |
| hsa-miR-26a-5p | 0 |
| hsa-miR-26b-3p | 4.80 |
| hsa-miR-27b-3p | 2.19 |
| hsa-miR-29c-3p | 2.61 |
| hsa-miR-30a-5p | 77.69 |
| hsa-miR-30d-5p | 0 |
| hsa-miR-31-5p | 78.42 |
| hsa-miR-340-3p | 62.57 |
| hsa-miR-342-3p | 0 |
| hsa-miR-361-5p | 0.31 |
| hsa-miR-363-3p | 0.21 |
| hsa-miR-425-5p | 0 |
| hsa-miR-454-3p | 1.36 |
| hsa-miR-455-5p | 98.33 |
| hsa-miR-483-3p | 86.34 |
| hsa-miR-5001-3p | 69.97 |
| hsa-miR-501-3p | 3.75 |
| hsa-miR-550a-3p | 0.10 |
| hsa-miR-671-3p | 71.95 |
| hsa-miR-885-5p | 96.25 |
| hsa-miR-92a-3p | 0 |
| hsa-miR-93-5p | 0 |
| hsa-miR-98-5p | 26.38 |
| UniSp3 | 0.31 |
| UniSp6 | 0 |

Legend: removed markers are in bold

**eTable 2 : detailed view of the bioinformatics tools used**

| **Tool** | **Reference** |
| --- | --- |
| EnrichmentMap | Merico D, Isserlin R, Stueker O, Emili A, Bader GD. Enrichment Map: A Network-Based Method for Gene-Set Enrichment Visualization and Interpretation. PLOS ONE. 2010 Nov 15;5(11):e13984. |
| G:Profiler | Kolberg L, Raudvere U, Kuzmin I, Vilo J, Peterson H. gprofiler2 -- an R package for gene list functional enrichment analysis and namespace conversion toolset g:Profiler [Internet]. F1000Research; 2020 [cited 2023 Mar 12]. Available from: <https://f1000research.com/articles/9-709> |
| miRTarbase | Huang HY, Lin YCD, Li J, Huang KY, Shrestha S, Hong HC, et al. miRTarBase 2020: updates to the experimentally validated microRNA–target interaction database. Nucleic Acids Research. 2019;48(D1):D148–54. |
| UpSetR | Conway JR, Lex A, Gehlenborg N. UpSetR: an R package for the visualization of intersecting sets and their properties. Bioinformatics (Oxford, England). 2017/06/25 ed. 2017 Sep 15;33(18):2938–40. |
| BRAINEAC | Ramasamy A, Trabzuni D, Guelfi S, Varghese V, Smith C, Walker R, et al. Genetic variability in the regulation of gene expression in ten regions of the human brain. Nature neuroscience. 2014/09/01 ed. 2014 Oct;17(10):1418–28. |
| HaploReg | Ward LD, Kellis M. HaploReg v4: systematic mining of putative causal variants, cell types, regulators and target genes for human complex traits and disease. Nucleic Acids Res. 2016 Jan 4;44(D1):D877-881. |
| ENCODE | Davis CA, Hitz BC, Sloan CA, Chan ET, Davidson JM, Gabdank I, et al. The Encyclopedia of DNA elements (ENCODE): data portal update. Nucleic acids research. 2017/11/11 ed. 2018 Jan 4;46(D1):D794-d801. |
| VARadb | Pan Q, Liu YJ, Bai XF, Han XL, Jiang Y, Ai B, et al. VARAdb: a comprehensive variation annotation database for human. Nucleic Acids Research. 2021 Jan 8;49(D1):D1431–44. |
| Haploview | Barrett JC, Fry B, Maller J, Daly MJ. Haploview: analysis and visualization of LD and haplotype maps. Bioinformatics (Oxford, England). 2004/08/07 ed. 2005 Jan 15;21(2):263–5. |

**eTable 3: Regression coefficient with RBANS as outcome variable and miRNA normalised Ct value as predictor, model adjusted for age, gender, education years, ethnicity, APOE ε4 carrier status**

| **domain** | **miRNA** | **b** | **[95% CI]** | **Std. Error** | **t value** | **P value** | **FDR adj.**  **P value** |
| --- | --- | --- | --- | --- | --- | --- | --- |
| **Language Index** | hsa.let.7a.5p | -2.39 | [-4.10, -0.69] | 0.868 | -2.752 | 0.006 | 0.048 |
|  | hsa.let.7c.5p | -2.26 | [-3.97, -0.56] | 0.871 | -2.601 | 0.009 | 0.049 |
|  | hsa.let.7d.5p | -3.06 | [-1.67, 1.75] | 1.023 | -2.992 | 0.003 | 0.048 |
|  | hsa.miR.144.5p | -0.89 | [-1.53, -0.25] | 0.327 | -2.723 | 0.007 | 0.048 |
|  | hsa.miR.93.5p | -1.88 | [-3.31, -0.45] | 0.728 | -2.577 | 0.01 | 0.049 |
|  | hsa.miR.98.5p | -1.95 | [-3.34, -0.55] | 0.712 | -2.735 | 0.006 | 0.048 |
| **Attention Index** | hsa.miR.363.3p | -4.27 | [-6.70, -1.85] | 1.236 | -3.457 | 0.001 | 0.017 |
| **Total Scale** | hsa.miR.144.5p | -1.17 | [-1.84, -0.51] | 0.339 | -3.464 | 0.001 | 0.016 |
| Legend: b = beta coefficient, CI = confidence interval, Std. Error = Standard Error | | | | | | | |

**eTable 4: Correlation matrix for the six significant miRNAs**

| **rho (spearman)** | |  |  |  |  |  |
| --- | --- | --- | --- | --- | --- | --- |
|  | miR.128.3p | miR.144.5p | miR.146a.5p | miR.26a.5p | miR.29c.3p | miR.363.3p |
| miR.128.3p | 1 | 0.09 | 0.27 | 0.33 | 0.15 | 0.26 |
| miR.144.5p | 0.09 | 1 | 0.38 | 0.43 | 0.45 | 0.35 |
| miR.146a.5p | 0.27 | 0.38 | 1 | 0.42 | 0.46 | 0.46 |
| miR.26a.5p | 0.33 | 0.43 | 0.42 | 1 | 0.41 | 0.52 |
| miR.29c.3p | 0.15 | 0.45 | 0.46 | 0.41 | 1 | 0.55 |
| miR.363.3p | 0.26 | 0.35 | 0.46 | 0.52 | 0.55 | 1 |
|  |  |  |  |  |  |  |
| **N** |  |  |  |  |  |  |
|  | miR.128.3p | miR.144.5p | miR.146a.5p | miR.26a.5p | miR.29c.3p | miR.363.3p |
| miR.128.3p | 818 | 798 | 804 | 805 | 799 | 804 |
| miR.144.5p | 798 | 810 | 796 | 802 | 795 | 798 |
| miR.146a.5p | 804 | 796 | 815 | 801 | 798 | 801 |
| miR.26a.5p | 805 | 802 | 801 | 816 | 798 | 804 |
| miR.29c.3p | 799 | 795 | 798 | 798 | 811 | 798 |
| miR.363.3p | 804 | 798 | 801 | 804 | 798 | 815 |
|  |  |  |  |  |  |  |
| **P values** |  |  |  |  |  |  |
|  | miR.128.3p | miR.144.5p | miR.146a.5p | miR.26a.5p | miR.29c.3p | miR.363.3p |
| miR.128.3p |  | 0.0128 | 0 | 0 | 0 | 0 |
| miR.144.5p | 0.0128 |  | 0 | 0 | 0 | 0 |
| miR.146a.5p | <0.0001 | <0.0001 |  | 0 | 0 | 0 |
| miR.26a.5p | <0.0001 | <0.0001 | <0.0001 |  | 0 | 0 |
| miR.29c.3p | <0.0001 | <0.0001 | <0.0001 | <0.0001 |  | 0 |
| miR.363.3p | <0.0001 | <0.0001 | <0.0001 | <0.0001 | <0.0001 |  |

**eFigure 1: Correlation matrix for the significant miRNAs**


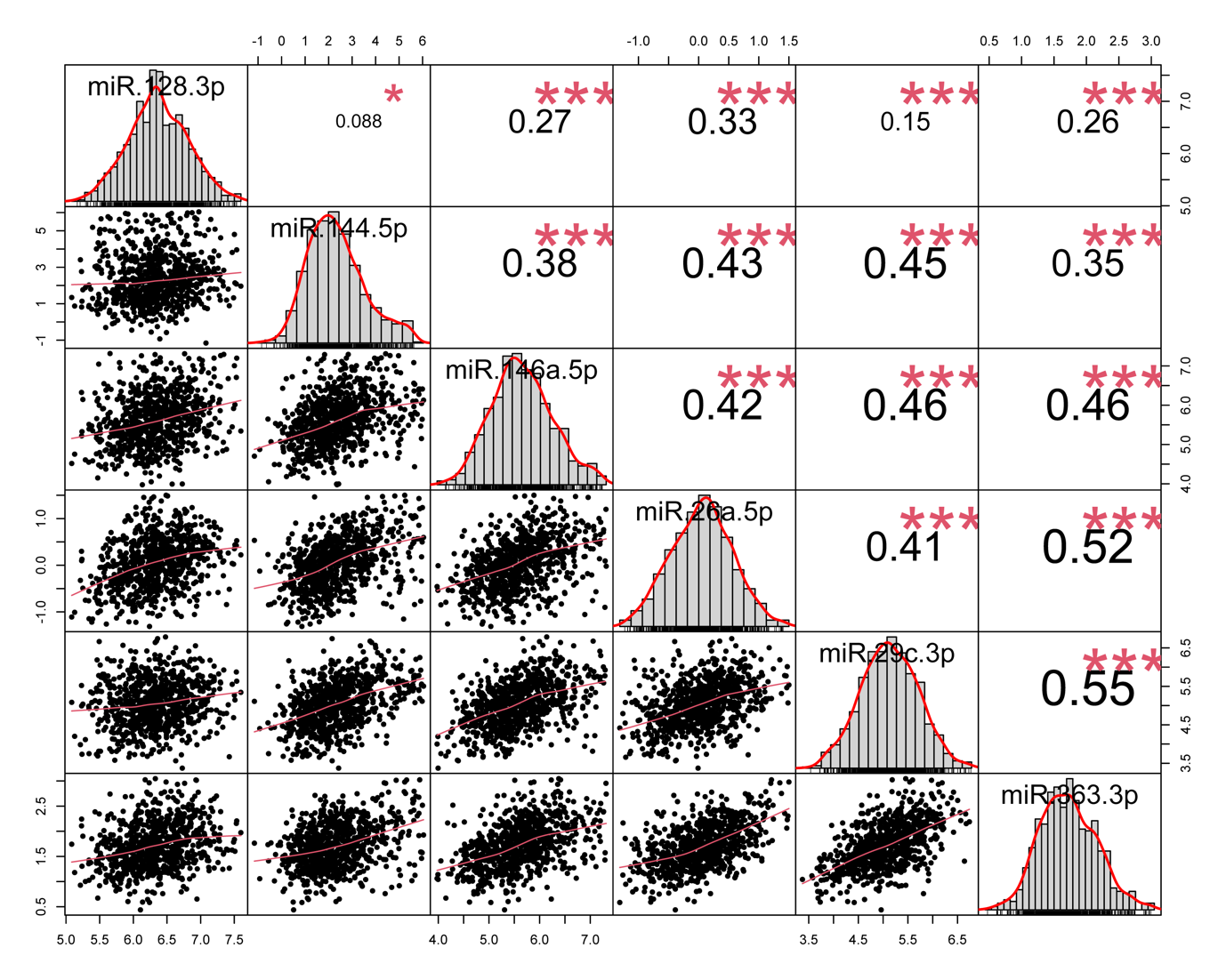


**eTable 5: Pathway enrichment analysis of the six significantly dysregulated miRNAs in the blood for targeted genes highly expressed in the brain**

| **ID** | **Description** | **FDR** | **intersection** | **cluster** | **mirnas** | **N mirnas** |
| --- | --- | --- | --- | --- | --- | --- |
| GO:1905710 | positive regulation of membrane permeability | 0.005 | GSK3B,SLC25A5,YWHAE,BLOC1S2 | cellular architecture | hsa-miR-26a-5p | 1 |
| REAC:R-HSA-975110 | TRAF6 mediated IRF7 activation in TLR7/8 or 9 signaling | 0.006 | IRAK1,IRF7 | toll like receptor signaling pathway | hsa-miR-146a-5p | 1 |
| GO:0006476 | protein deacetylation | 0.006 | RCOR1,PHB,SFPQ,MTA3 | protein synthesis | hsa-miR-26a-5p | 1 |
| GO:0090559 | regulation of membrane permeability | 0.006 | GSK3B,SLC25A5,YWHAE,BLOC1S2 | cellular architecture | hsa-miR-26a-5p | 1 |
| REAC:R-HSA-9006925 | Intracellular signaling by second messengers | 0.007 | GSK3B,RPS27A,TNRC6B,RCOR1,MDM2,PPP2R5D,MTA3,PRKX | cellular signaling | hsa-miR-146a-5p, hsa-miR-146a-5p, hsa-miR-26a-5p, hsa-miR-26a-5p | 4 |
| REAC:R-HSA-9006925 | Intracellular signaling by second messengers | 0.007 | GSK3B,RPS27A,TNRC6B,RCOR1,MDM2,PPP2R5D,MTA3,PRKX | cellular signaling | hsa-miR-146a-5p, hsa-miR-146a-5p, hsa-miR-26a-5p, hsa-miR-26a-5p | 4 |
| GO:0098732 | macromolecule deacylation | 0.007 | RCOR1,PHB,SFPQ,MTA3 | protein synthesis | hsa-miR-26a-5p | 1 |
| GO:1905214 | regulation of RNA binding | 0.009 | CDK9,NUCKS1 | transcription and splicing | hsa-miR-26a-5p | 1 |
| REAC:R-HSA-1257604 | PIP3 activates AKT signaling | 0.009 | GSK3B,RPS27A,TNRC6B,RCOR1,MDM2,PPP2R5D,MTA3 | tyrosine kinase signaling | hsa-miR-26a-5p | 1 |
| REAC:R-HSA-212165 | Epigenetic regulation of gene expression | 0.009 | GSK3B,UBTF,TDG,MTA3,POLR2E | epigenetic changes | hsa-miR-26a-5p | 1 |
| REAC:R-HSA-3700989 | Transcriptional Regulation by TP53 | 0.009 | CCNE1,RPS27A,YWHAE,TNRC6B,MDM2,CDK9,POLR2E,COX5A | transcription and splicing | hsa-miR-26a-5p, hsa-miR-29c-3p | 2 |
| GO:0016032 | viral process | 0.013 | RPS27A,DDB1,PDE12,PHB,CDK9,POLR2E,NUCKS1 | viral processes | hsa-miR-26a-5p | 1 |
| GO:0031570 | DNA integrity checkpoint | 0.014 | MDM2,CNOT4,CDC5L,CNOT2 | cell cycle | hsa-miR-363-3p | 1 |
| GO:0044783 | G1 DNA damage checkpoint | 0.015 | MDM2,CNOT4,CNOT2 | cell cycle | hsa-miR-363-3p | 1 |
| REAC:R-HSA-198323 | AKT phosphorylates targets in the cytosol | 0.017 | AKT3,MDM2 | cellular signaling | hsa-miR-26a-5p, hsa-miR-29c-3p | 2 |
| REAC:R-HSA-6804759 | Regulation of TP53 Activity through Association with Co-factors | 0.017 | PPP1R13B,AKT3 | cellular signaling | hsa-miR-29c-3p | 1 |
| GO:0006283 | transcription-coupled nucleotide-excision repair | 0.021 | RPS27A,DDB1,POLR2E | cell cycle | hsa-miR-26a-5p | 1 |
| GO:0090305 | nucleic acid phosphodiester bond hydrolysis | 0.021 | RPS27A,DDB1,PDE12,CPSF2 | cell cycle | hsa-miR-26a-5p | 1 |
| GO:0019058 | viral life cycle | 0.023 | RPS27A,DDB1,PDE12,PHB,NUCKS1 | viral processes | hsa-miR-26a-5p | 1 |
| REAC:R-HSA-8849470 | PTK6 Regulates Cell Cycle | 0.024 | CCNE1 | cell cycle | hsa-miR-144-5p | 1 |
| GO:0046677 | response to antibiotic | 0.026 | MDM2,RPL23 | response to external stimuli | hsa-miR-363-3p | 1 |
| REAC:R-HSA-166166 | MyD88-independent TLR4 cascade | 0.026 | IRAK1,IRF7 | toll like receptor signaling pathway | hsa-miR-146a-5p | 1 |
| REAC:R-HSA-168138 | Toll Like Receptor 9 (TLR9) Cascade | 0.026 | IRAK1,IRF7 | toll like receptor signaling pathway | hsa-miR-146a-5p | 1 |
| REAC:R-HSA-168164 | Toll Like Receptor 3 (TLR3) Cascade | 0.026 | IRAK1,IRF7 | toll like receptor signaling pathway | hsa-miR-146a-5p | 1 |
| REAC:R-HSA-168181 | Toll Like Receptor 7/8 (TLR7/8) Cascade | 0.026 | IRAK1,IRF7 | toll like receptor signaling pathway | hsa-miR-146a-5p | 1 |
| REAC:R-HSA-9006925 | Intracellular signaling by second messengers | 0.026 | IRAK1,MTA2,PRKCE | cellular signaling | hsa-miR-146a-5p, hsa-miR-146a-5p, hsa-miR-26a-5p, hsa-miR-26a-5p | 4 |
| REAC:R-HSA-9006925 | Intracellular signaling by second messengers | 0.026 | IRAK1,MTA2,PRKCE | cellular signaling | hsa-miR-146a-5p, hsa-miR-146a-5p, hsa-miR-26a-5p, hsa-miR-26a-5p | 4 |
| REAC:R-HSA-937061 | TRIF(TICAM1)-mediated TLR4 signaling | 0.026 | IRAK1,IRF7 | toll like receptor signaling pathway | hsa-miR-146a-5p | 1 |
| GO:0000956 | nuclear-transcribed mRNA catabolic process | 0.027 | CNOT4,CNOT2,RPL23,RPL24 | cell cycle | hsa-miR-363-3p | 1 |
| GO:0002221 | pattern recognition receptor signaling pathway | 0.029 | IRAK1,IRF7,PRKCE | cellular signaling | hsa-miR-146a-5p | 1 |
| GO:0002224 | toll-like receptor signaling pathway | 0.029 | IRAK1,IRF7,PRKCE | toll like receptor signaling pathway | hsa-miR-146a-5p | 1 |
| GO:0002755 | MyD88-dependent toll-like receptor signaling pathway | 0.029 | IRAK1,IRF7 | toll like receptor signaling pathway | hsa-miR-146a-5p | 1 |
| GO:0002756 | MyD88-independent toll-like receptor signaling pathway | 0.029 | IRF7,PRKCE | toll like receptor signaling pathway | hsa-miR-146a-5p | 1 |
| GO:0034142 | toll-like receptor 4 signaling pathway | 0.029 | IRAK1,PRKCE | toll like receptor signaling pathway | hsa-miR-146a-5p | 1 |
| REAC:R-HSA-166016 | Toll Like Receptor 4 (TLR4) Cascade | 0.03 | IRAK1,IRF7 | toll like receptor signaling pathway | hsa-miR-146a-5p | 1 |
| GO:0051702 | biological process involved in interaction with symbiont | 0.031 | DDB1,PHB,NUCKS1 | cellular signaling | hsa-miR-26a-5p | 1 |
| REAC:R-HSA-110357 | Displacement of DNA glycosylase by APEX1 | 0.033 | MBD4 | cell cycle | hsa-miR-146a-5p | 1 |
| REAC:R-HSA-168898 | Toll-like Receptor Cascades | 0.033 | IRAK1,IRF7 | toll like receptor signaling pathway | hsa-miR-146a-5p | 1 |
| REAC:R-HSA-3134963 | DEx/H-box helicases activate type I IFN and inflammatory cytokines production | 0.033 | IRF7 | cytokine | hsa-miR-146a-5p | 1 |
| REAC:R-HSA-3304351 | Signaling by TGF-beta Receptor Complex in Cancer | 0.033 | SMAD2 | cytokine | hsa-miR-146a-5p | 1 |
| REAC:R-HSA-2559585 | Oncogene Induced Senescence | 0.033 | RPS27A,TNRC6B,MDM2 | senescence | hsa-miR-26a-5p | 1 |
| GO:0019080 | viral gene expression | 0.033 | RPS27A,CDK9,POLR2E,NUCKS1 | viral processes | hsa-miR-26a-5p | 1 |
| GO:0070911 | global genome nucleotide-excision repair | 0.033 | RPS27A,DDB1 | cell cycle | hsa-miR-26a-5p | 1 |
| REAC:R-HSA-114452 | Activation of BH3-only proteins | 0.034 | PPP1R13B,AKT3 | apoptosis and senescence | hsa-miR-29c-3p | 1 |
| REAC:R-HSA-2173796 | SMAD2/SMAD3:SMAD4 heterotrimer regulates transcription | 0.034 | CCNT2,WWTR1 | transcription and splicing | hsa-miR-29c-3p | 1 |
| REAC:R-HSA-3700989 | Transcriptional Regulation by TP53 | 0.034 | PPP1R13B,AKT3,CCNT2,MDM2 | transcription and splicing | hsa-miR-26a-5p, hsa-miR-29c-3p | 2 |
| REAC:R-HSA-5633007 | Regulation of TP53 Activity | 0.034 | PPP1R13B,AKT3,MDM2 | apoptosis and senescence | hsa-miR-29c-3p | 1 |
| REAC:R-HSA-5674400 | Constitutive Signaling by AKT1 E17K in Cancer | 0.034 | AKT3,MDM2 | signaling activity | hsa-miR-29c-3p | 1 |
| REAC:R-HSA-6804757 | Regulation of TP53 Degradation | 0.034 | AKT3,MDM2 | apoptosis and senescence | hsa-miR-29c-3p | 1 |
| REAC:R-HSA-6806003 | Regulation of TP53 Expression and Degradation | 0.034 | AKT3,MDM2 | apoptosis and senescence | hsa-miR-29c-3p | 1 |
| REAC:R-HSA-1538133 | G0 and Early G1 | 0.034 | CCNE1 | cell cycle | hsa-miR-144-5p | 1 |
| REAC:R-HSA-1638091 | Heparan sulfate/heparin (HS-GAG) metabolism | 0.034 | HS3ST1 | hormone and metabolites | hsa-miR-144-5p | 1 |
| REAC:R-HSA-2022928 | HS-GAG biosynthesis | 0.034 | HS3ST1 | hormone and metabolites | hsa-miR-144-5p | 1 |
| REAC:R-HSA-2559586 | DNA Damage/Telomere Stress Induced Senescence | 0.034 | CCNE1 | apoptosis and senescence | hsa-miR-144-5p | 1 |
| REAC:R-HSA-390471 | Association of TriC/CCT with target proteins during biosynthesis | 0.034 | CCNE1 | protein synthesis | hsa-miR-144-5p | 1 |
| REAC:R-HSA-6791312 | TP53 Regulates Transcription of Cell Cycle Genes | 0.034 | CCNE1 | cell cycle | hsa-miR-144-5p | 1 |
| REAC:R-HSA-6804116 | TP53 Regulates Transcription of Genes Involved in G1 Cell Cycle Arrest | 0.034 | CCNE1 | cell cycle | hsa-miR-144-5p | 1 |
| REAC:R-HSA-69017 | CDK-mediated phosphorylation and removal of Cdc6 | 0.034 | CCNE1 | cell cycle | hsa-miR-144-5p | 1 |
| REAC:R-HSA-69205 | G1/S-Specific Transcription | 0.034 | CCNE1 | cell cycle | hsa-miR-144-5p | 1 |
| REAC:R-HSA-69563 | p53-Dependent G1 DNA Damage Response | 0.034 | CCNE1 | cell cycle | hsa-miR-144-5p, hsa-miR-144-5p, hsa-miR-26a-5p, hsa-miR-26a-5p | 4 |
| REAC:R-HSA-69563 | p53-Dependent G1 DNA Damage Response | 0.034 | CCNE1 | cell cycle | hsa-miR-144-5p, hsa-miR-144-5p, hsa-miR-26a-5p, hsa-miR-26a-5p | 4 |
| REAC:R-HSA-69580 | p53-Dependent G1/S DNA damage checkpoint | 0.034 | CCNE1 | cell cycle | hsa-miR-144-5p, hsa-miR-144-5p, hsa-miR-26a-5p, hsa-miR-26a-5p | 4 |
| REAC:R-HSA-69580 | p53-Dependent G1/S DNA damage checkpoint | 0.034 | CCNE1 | cell cycle | hsa-miR-144-5p, hsa-miR-144-5p, hsa-miR-26a-5p, hsa-miR-26a-5p | 4 |
| REAC:R-HSA-69615 | G1/S DNA Damage Checkpoints | 0.034 | CCNE1 | cell cycle | hsa-miR-144-5p, hsa-miR-144-5p, hsa-miR-26a-5p, hsa-miR-26a-5p | 4 |
| REAC:R-HSA-69615 | G1/S DNA Damage Checkpoints | 0.034 | CCNE1 | cell cycle | hsa-miR-144-5p, hsa-miR-144-5p, hsa-miR-26a-5p, hsa-miR-26a-5p | 4 |
| REAC:R-HSA-8848021 | Signaling by PTK6 | 0.034 | CCNE1 | tyrosine kinase signaling | hsa-miR-144-5p, hsa-miR-144-5p, hsa-miR-26a-5p, hsa-miR-26a-5p | 4 |
| REAC:R-HSA-8848021 | Signaling by PTK6 | 0.034 | CCNE1 | tyrosine kinase signaling | hsa-miR-144-5p, hsa-miR-144-5p, hsa-miR-26a-5p, hsa-miR-26a-5p | 4 |
| REAC:R-HSA-9006927 | Signaling by Non-Receptor Tyrosine Kinases | 0.034 | CCNE1 | tyrosine kinase signaling | hsa-miR-144-5p, hsa-miR-144-5p, hsa-miR-26a-5p, hsa-miR-26a-5p | 4 |
| REAC:R-HSA-9006927 | Signaling by Non-Receptor Tyrosine Kinases | 0.034 | CCNE1 | tyrosine kinase signaling | hsa-miR-144-5p, hsa-miR-144-5p, hsa-miR-26a-5p, hsa-miR-26a-5p | 4 |
| GO:0016311 | dephosphorylation | 0.036 | GSK3B,YWHAE,PTPN13,PPP2R5D,MTMR12 | cellular signaling | hsa-miR-26a-5p | 1 |
| REAC:R-HSA-69202 | Cyclin E associated events during G1/S transition | 0.036 | CCNE1 | cell cycle | hsa-miR-144-5p | 1 |
| REAC:R-HSA-69656 | Cyclin A:Cdk2-associated events at S phase entry | 0.036 | CCNE1 | cell cycle | hsa-miR-144-5p | 1 |
| REAC:R-HSA-69052 | Switching of origins to a post-replicative state | 0.037 | CCNE1 | cell cycle | hsa-miR-144-5p | 1 |
| REAC:R-HSA-390466 | Chaperonin-mediated protein folding | 0.037 | CCNE1 | protein synthesis | hsa-miR-144-5p | 1 |
| REAC:R-HSA-112382 | Formation of RNA Pol II elongation complex | 0.037 | RTF1,CDK9,POLR2E | transcription and splicing | hsa-miR-26a-5p | 1 |
| REAC:R-HSA-198323 | AKT phosphorylates targets in the cytosol | 0.037 | GSK3B,MDM2 | cellular signaling | hsa-miR-26a-5p, hsa-miR-29c-3p | 2 |
| REAC:R-HSA-4839735 | Signaling by AXIN mutants | 0.037 | GSK3B,PPP2R5D | Wnt/beta-catenin signaling pathway | hsa-miR-26a-5p | 1 |
| REAC:R-HSA-4839743 | Signaling by CTNNB1 phospho-site mutants | 0.037 | GSK3B,PPP2R5D | Wnt/beta-catenin signaling pathway | hsa-miR-26a-5p | 1 |
| REAC:R-HSA-4839744 | Signaling by APC mutants | 0.037 | GSK3B,PPP2R5D | Wnt/beta-catenin signaling pathway | hsa-miR-26a-5p | 1 |
| REAC:R-HSA-4839748 | Signaling by AMER1 mutants | 0.037 | GSK3B,PPP2R5D | Wnt/beta-catenin signaling pathway | hsa-miR-26a-5p | 1 |
| REAC:R-HSA-5339716 | Signaling by GSK3beta mutants | 0.037 | GSK3B,PPP2R5D | Wnt/beta-catenin signaling pathway | hsa-miR-26a-5p | 1 |
| REAC:R-HSA-5358747 | S33 mutants of beta-catenin aren't phosphorylated | 0.037 | GSK3B,PPP2R5D | Wnt/beta-catenin signaling pathway | hsa-miR-26a-5p | 1 |
| REAC:R-HSA-5358749 | S37 mutants of beta-catenin aren't phosphorylated | 0.037 | GSK3B,PPP2R5D | Wnt/beta-catenin signaling pathway | hsa-miR-26a-5p | 1 |
| REAC:R-HSA-5358751 | S45 mutants of beta-catenin aren't phosphorylated | 0.037 | GSK3B,PPP2R5D | Wnt/beta-catenin signaling pathway | hsa-miR-26a-5p | 1 |
| REAC:R-HSA-5358752 | T41 mutants of beta-catenin aren't phosphorylated | 0.037 | GSK3B,PPP2R5D | Wnt/beta-catenin signaling pathway | hsa-miR-26a-5p | 1 |
| REAC:R-HSA-5467337 | APC truncation mutants have impaired AXIN binding | 0.037 | GSK3B,PPP2R5D | Wnt/beta-catenin signaling pathway | hsa-miR-26a-5p | 1 |
| REAC:R-HSA-5467340 | AXIN missense mutants destabilize the destruction complex | 0.037 | GSK3B,PPP2R5D | Wnt/beta-catenin signaling pathway | hsa-miR-26a-5p | 1 |
| REAC:R-HSA-5467348 | Truncations of AMER1 destabilize the destruction complex | 0.037 | GSK3B,PPP2R5D | Wnt/beta-catenin signaling pathway | hsa-miR-26a-5p | 1 |
| REAC:R-HSA-6781823 | Formation of TC-NER Pre-Incision Complex | 0.037 | RPS27A,DDB1,POLR2E | cell cycle | hsa-miR-26a-5p | 1 |
| REAC:R-HSA-73762 | RNA Polymerase I Transcription Initiation | 0.037 | UBTF,MTA3,POLR2E | transcription and splicing | hsa-miR-26a-5p | 1 |
| REAC:R-HSA-75955 | RNA Polymerase II Transcription Elongation | 0.037 | RTF1,CDK9,POLR2E | transcription and splicing | hsa-miR-26a-5p | 1 |
| REAC:R-HSA-8848021 | Signaling by PTK6 | 0.037 | CCNE1,RPS27A,SFPQ | tyrosine kinase signaling | hsa-miR-144-5p, hsa-miR-144-5p, hsa-miR-26a-5p, hsa-miR-26a-5p | 4 |
| REAC:R-HSA-8848021 | Signaling by PTK6 | 0.037 | CCNE1,RPS27A,SFPQ | tyrosine kinase signaling | hsa-miR-144-5p, hsa-miR-144-5p, hsa-miR-26a-5p, hsa-miR-26a-5p | 4 |
| REAC:R-HSA-9006927 | Signaling by Non-Receptor Tyrosine Kinases | 0.037 | CCNE1,RPS27A,SFPQ | tyrosine kinase signaling | hsa-miR-144-5p, hsa-miR-144-5p, hsa-miR-26a-5p, hsa-miR-26a-5p | 4 |
| REAC:R-HSA-9006927 | Signaling by Non-Receptor Tyrosine Kinases | 0.037 | CCNE1,RPS27A,SFPQ | tyrosine kinase signaling | hsa-miR-144-5p, hsa-miR-144-5p, hsa-miR-26a-5p, hsa-miR-26a-5p | 4 |
| REAC:R-HSA-391251 | Protein folding | 0.037 | CCNE1 | protein synthesis | hsa-miR-144-5p | 1 |
| REAC:R-HSA-2468052 | Establishment of Sister Chromatid Cohesion | 0.038 | STAG1,PDS5A | cell cycle | hsa-miR-128-3p | 1 |
| REAC:R-HSA-2470946 | Cohesin Loading onto Chromatin | 0.038 | STAG1,PDS5A | cell cycle | hsa-miR-128-3p | 1 |
| REAC:R-HSA-2555396 | Mitotic Metaphase and Anaphase | 0.038 | STAG1,LMNB1,KPNB1,PDS5A,TNPO1 | cell cycle | hsa-miR-128-3p | 1 |
| REAC:R-HSA-447115 | Interleukin-12 family signaling | 0.038 | LMNB1,HNRNPF,CANX | cytokine | hsa-miR-128-3p | 1 |
| REAC:R-HSA-68882 | Mitotic Anaphase | 0.038 | STAG1,LMNB1,KPNB1,PDS5A,TNPO1 | cell cycle | hsa-miR-128-3p | 1 |
| REAC:R-HSA-68884 | Mitotic Telophase/Cytokinesis | 0.038 | STAG1,PDS5A | cell cycle | hsa-miR-128-3p | 1 |
| GO:0044788 | modulation by host of viral process | 0.039 | PHB,NUCKS1 | viral processes | hsa-miR-26a-5p | 1 |
| GO:0072331 | signal transduction by p53 class mediator | 0.04 | MDM2,CNOT4,CNOT2,RPL23 | cell cycle | hsa-miR-363-3p | 1 |
| GO:0097168 | mesenchymal stem cell proliferation | 0.041 | CCNE1 | cell cycle | hsa-miR-144-5p | 1 |
| REAC:R-HSA-1630316 | Glycosaminoglycan metabolism | 0.041 | HS3ST1 | hormone and metabolites | hsa-miR-144-5p | 1 |
| REAC:R-HSA-69206 | G1/S Transition | 0.041 | CCNE1 | cell cycle | hsa-miR-144-5p | 1 |
| REAC:R-HSA-69306 | DNA Replication | 0.041 | CCNE1 | cell cycle | hsa-miR-144-5p | 1 |
| REAC:R-HSA-196299 | Beta-catenin phosphorylation cascade | 0.041 | GSK3B,PPP2R5D | Wnt/beta-catenin signaling pathway | hsa-miR-26a-5p | 1 |
| REAC:R-HSA-6782135 | Dual incision in TC-NER | 0.041 | RPS27A,DDB1,POLR2E | cell cycle | hsa-miR-26a-5p | 1 |
| REAC:R-HSA-6782210 | Gap-filling DNA repair synthesis and ligation in TC-NER | 0.041 | RPS27A,DDB1,POLR2E | cell cycle | hsa-miR-26a-5p | 1 |
| REAC:R-HSA-6807070 | PTEN Regulation | 0.041 | RPS27A,TNRC6B,RCOR1,MTA3 | cell cycle | hsa-miR-26a-5p | 1 |
| REAC:R-HSA-69563 | p53-Dependent G1 DNA Damage Response | 0.041 | CCNE1,RPS27A,MDM2 | cell cycle | hsa-miR-144-5p, hsa-miR-144-5p, hsa-miR-26a-5p, hsa-miR-26a-5p | 4 |
| REAC:R-HSA-69563 | p53-Dependent G1 DNA Damage Response | 0.041 | CCNE1,RPS27A,MDM2 | cell cycle | hsa-miR-144-5p, hsa-miR-144-5p, hsa-miR-26a-5p, hsa-miR-26a-5p | 4 |
| REAC:R-HSA-69580 | p53-Dependent G1/S DNA damage checkpoint | 0.041 | CCNE1,RPS27A,MDM2 | cell cycle | hsa-miR-144-5p, hsa-miR-144-5p, hsa-miR-26a-5p, hsa-miR-26a-5p | 4 |
| REAC:R-HSA-69580 | p53-Dependent G1/S DNA damage checkpoint | 0.041 | CCNE1,RPS27A,MDM2 | cell cycle | hsa-miR-144-5p, hsa-miR-144-5p, hsa-miR-26a-5p, hsa-miR-26a-5p | 4 |
| GO:0032606 | type I interferon production | 0.041 | IRAK1,IRF7 | cytokine | hsa-miR-146a-5p | 1 |
| REAC:R-HSA-5663202 | Diseases of signal transduction by growth factor receptors and second messengers | 0.041 | GSK3B,RPS27A,PHB,MDM2,PPP2R5D,POLR2E | diseases | hsa-miR-26a-5p | 1 |
| REAC:R-HSA-69615 | G1/S DNA Damage Checkpoints | 0.041 | CCNE1,RPS27A,MDM2 | cell cycle | hsa-miR-144-5p, hsa-miR-144-5p, hsa-miR-26a-5p, hsa-miR-26a-5p | 4 |
| REAC:R-HSA-69615 | G1/S DNA Damage Checkpoints | 0.041 | CCNE1,RPS27A,MDM2 | cell cycle | hsa-miR-144-5p, hsa-miR-144-5p, hsa-miR-26a-5p, hsa-miR-26a-5p | 4 |
| REAC:R-HSA-5250913 | Positive epigenetic regulation of rRNA expression | 0.041 | GSK3B,MTA3,POLR2E | epigenetic changes | hsa-miR-26a-5p | 1 |
| GO:0000377 | RNA splicing, via transesterification reactions with bulged adenosine as nucleophile | 0.041 | HNRNPU,SFPQ,POLR2E,HNRNPA0,CPSF2 | transcription and splicing | hsa-miR-26a-5p | 1 |
| GO:0006289 | nucleotide-excision repair | 0.041 | RPS27A,DDB1,POLR2E | cell cycle | hsa-miR-26a-5p | 1 |
| REAC:R-HSA-6804760 | Regulation of TP53 Activity through Methylation | 0.041 | RPS27A,MDM2 | epigenetic changes | hsa-miR-26a-5p | 1 |
| GO:0000375 | RNA splicing, via transesterification reactions | 0.042 | HNRNPU,SFPQ,POLR2E,HNRNPA0,CPSF2 | transcription and splicing | hsa-miR-26a-5p | 1 |
| KEGG:04110 | Cell cycle | 0.043 | GSK3B,CCNE1,YWHAE,MDM2 | cell cycle | hsa-miR-26a-5p | 1 |
| KEGG:04120 | Ubiquitin mediated proteolysis | 0.043 | UBA2,RPS27A,DDB1,MDM2 | protein synthesis | hsa-miR-26a-5p | 1 |
| REAC:R-HSA-109606 | Intrinsic Pathway for Apoptosis | 0.044 | PPP1R13B,AKT3 | apoptosis and senescence | hsa-miR-29c-3p | 1 |
| REAC:R-HSA-202131 | Metabolism of nitric oxide: NOS3 activation and regulation | 0.044 | WASL,DDAH1 | cellular signaling | hsa-miR-128-3p | 1 |
| GO:0031109 | microtubule polymerization or depolymerization | 0.044 | ZNF207,BLOC1S2,TUBGCP5 | cellular architecture | hsa-miR-26a-5p | 1 |
| GO:0044843 | cell cycle G1/S phase transition | 0.044 | MDM2,CNOT4,CNOT2,FBXW7 | cell cycle | hsa-miR-363-3p | 1 |
| REAC:R-HSA-453279 | Mitotic G1 phase and G1/S transition | 0.044 | CCNE1 | cell cycle | hsa-miR-144-5p | 1 |
| REAC:R-HSA-2995383 | Initiation of Nuclear Envelope (NE) Reformation | 0.045 | LMNB1,KPNB1 | cell cycle | hsa-miR-128-3p | 1 |
| REAC:R-HSA-2559583 | Cellular Senescence | 0.045 | CCNE1 | senescence | hsa-miR-144-5p, hsa-miR-26a-5p | 2 |
| REAC:R-HSA-69242 | S Phase | 0.045 | CCNE1 | cell cycle | hsa-miR-144-5p | 1 |
| KEGG:04064 | NF-kappa B signaling pathway | 0.046 | IRAK1,CARD10 | NF kappa beta signaling pathway | hsa-miR-146a-5p | 1 |
| KEGG:04620 | Toll-like receptor signaling pathway | 0.046 | IRAK1,IRF7 | toll like receptor signaling pathway | hsa-miR-146a-5p | 1 |
| KEGG:04933 | AGE-RAGE signaling pathway in diabetic complications | 0.046 | SMAD2,PRKCE | signaling activity | hsa-miR-146a-5p | 1 |
| KEGG:05142 | Chagas disease | 0.046 | IRAK1,SMAD2 | diseases | hsa-miR-146a-5p | 1 |
| REAC:R-HSA-73854 | RNA Polymerase I Promoter Clearance | 0.047 | UBTF,MTA3,POLR2E | cell cycle | hsa-miR-26a-5p | 1 |
| REAC:R-HSA-73864 | RNA Polymerase I Transcription | 0.047 | UBTF,MTA3,POLR2E | cell cycle | hsa-miR-26a-5p | 1 |
| REAC:R-HSA-2559583 | Cellular Senescence | 0.047 | CCNE1,RPS27A,TNRC6B,MDM2 | senescence | hsa-miR-144-5p, hsa-miR-26a-5p | 2 |
| REAC:R-HSA-1502540 | Signaling by Activin | 0.048 | SMAD2 | hormones | hsa-miR-146a-5p | 1 |
| REAC:R-HSA-209543 | p75NTR recruits signalling complexes | 0.048 | IRAK1 | cellular signaling | hsa-miR-146a-5p | 1 |
| REAC:R-HSA-209560 | NF-kB is activated and signals survival | 0.048 | IRAK1 | nF kappa beta signaling pathway | hsa-miR-146a-5p | 1 |
| REAC:R-HSA-9013973 | TICAM1-dependent activation of IRF3/IRF7 | 0.048 | IRF7 | cellular signaling | hsa-miR-146a-5p | 1 |
| REAC:R-HSA-918233 | TRAF3-dependent IRF activation pathway | 0.048 | IRF7 | cellular signaling | hsa-miR-146a-5p | 1 |
| REAC:R-HSA-975144 | IRAK1 recruits IKK complex upon TLR7/8 or 9 stimulation | 0.048 | IRAK1 | toll like receptor signaling pathway | hsa-miR-146a-5p | 1 |
| GO:0034504 | protein localization to nucleus | 0.048 | GSK3B,HNRNPU,YWHAE,MDM2 | cellular architecture | hsa-miR-26a-5p | 1 |
| REAC:R-HSA-6781827 | Transcription-Coupled Nucleotide Excision Repair (TC-NER) | 0.048 | RPS27A,DDB1,POLR2E | transcription and splicing | hsa-miR-26a-5p | 1 |
| REAC:R-HSA-2995410 | Nuclear Envelope (NE) Reassembly | 0.05 | LMNB1,KPNB1,TNPO1 | cell cycle | hsa-miR-128-3p | 1 |
| GO:0006296 | nucleotide-excision repair, DNA incision, 5'-to lesion | 0.05 | RPS27A,DDB1 | cell cycle | hsa-miR-26a-5p | 1 |

**eTable 6 : Results from the miRNA gene association analysis including only cognitive normal individuals with positive ADRD CSF biomarkers.**

| ***microRNA***  ***gene*** | **biomarker** | **N participants** | **N**  **snps**  **(α_FDR_<0.05)** | **N**  **snps**  **(α_FDR_>0.05)** | **SNP list** |
| --- | --- | --- | --- | --- | --- |
| *MIR144* | pTau_181_ | 169 | 0 | 6 | rs9892942, rs901975, rs2874255, rs113539681, rs968726, rs138285687 |
| *MIR146* | t-tau | 169 | 0 | 10 | rs17057846, rs4921141, rs10075936, rs2910162, rs3096022, rs2431690, rs6878034, rs2431691, rs4921142, rs5010836 |
| *MIR26A* | BACE1 activity | 46 | 0 | 1 | rs73058983 |
| *MIR29C* | BACE1 activity | 46 | 0 | 5 | rs6669384, rs34599386, rs4844622, rs882198, rs12145290 |
| *MIR29C* | sTREM2 | 40 | 0 | 4 | rs34599386, rs4844622, rs882198, rs12145290 |
| *MIR29C* | t-tau | 169 | 0 | 7 | rs74821401, rs1204672, rs1204673, rs558248, rs2952, rs1555137, rs861475 |

**eTable 7: Role of selected brain specific transcription factors for which binding is affected by significant SNPs associated with Aβ42, BACE1 and sTREM2 levels in the CSF**

| **Transcription factor** | **Clinical significance** |
| --- | --- |
| Foxj2_2 | Forkhead transcription factors family are involved in neuronal death, neuronal response to amyloid ß exposure leading to mitochondrial dysfunction, pro-inflammatory cytokines production, apoptosis (1,2) |
| GATA_known1 | regulates SCNA transcription in dopaminergic neurons leading to increased levels of α-synuclein (3) |
| RXRA_known4 | variation in RXRa gene increases risk of AD through the regulation of genes involved in cholesterol metabolism (4) |
| SP1_disc3 | regulates expression of APP, tau, PSEN2 promoter transcription and BACE1 (5–10); in AD mouse model, inhibition of Sp1 increased memory deficits (11)25.04.23 09:51:00 |
| SP2_disc3 | involved in neurogenesis (12) |
| YY1_known6 | involved in neurogenesis in mice models (13) |

**References**

1. Maiese K. Forkhead transcription factors: new considerations for alzheimer’s disease and dementia. Journal of translational science. 2016/07/09 ed. 2016 Jul;2(4):241–7.

2. Maiese K. Forkhead Transcription Factors: Formulating a FOXO Target for Cognitive Loss. Current neurovascular research. 2017/11/19 ed. 2017;14(4):415–20.

3. Scherzer CR, Grass JA, Liao Z, Pepivani I, Zheng B, Eklund AC, et al. GATA transcription factors directly regulate the Parkinson’s disease-linked gene α-synuclein. Proceedings of the National Academy of Sciences. 2008;105(31):10907.

4. Kölsch H, Lütjohann D, Jessen F, Popp J, Hentschel F, Kelemen P, et al. RXRA gene variations influence Alzheimer’s disease risk and cholesterol metabolism. Journal of cellular and molecular medicine. 2009/04/21 ed. 2009 Mar;13(3):589–98.

5. Lukiw WJ, Rogaev EI, Wong L, Vaula G, McLachlan DR, St George Hyslop P. Protein-DNA interactions in the promoter region of the amyloid precursor protein (APP) gene in human neocortex. Brain research Molecular brain research. 1994/03/01 ed. 1994 Mar;22(1–4):121–31.

6. Querfurth HW, Jiang J, Xia W, Selkoe DJ. Enhancer function and novel DNA binding protein activity in the near upstream betaAPP gene promoter. Gene. 1999/05/20 ed. 1999 May 17;232(1):125–41.

7. Docagne F, Gabriel C, Lebeurrier N, Lesné S, Hommet Y, Plawinski L, et al. Sp1 and Smad transcription factors co-operate to mediate TGF-beta-dependent activation of amyloid-beta precursor protein gene transcription. The Biochemical journal. 2004/07/10 ed. 2004 Oct 15;383(Pt 2):393–9.

8. Christensen MA, Zhou W, Qing H, Lehman A, Philipsen S, Song W. Transcriptional regulation of BACE1, the beta-amyloid precursor protein beta-secretase, by Sp1. Molecular and cellular biology. 2004/01/01 ed. 2004 Jan;24(2):865–74.

9. Heicklen-Klein A, Ginzburg I. Tau promoter confers neuronal specificity and binds Sp1 and AP-2. Journal of neurochemistry. 2000/09/15 ed. 2000 Oct;75(4):1408–18.

10. Renbaum P, Beeri R, Gabai E, Amiel M, Gal M, Ehrengruber MU, et al. Egr-1 upregulates the Alzheimer’s disease presenilin-2 gene in neuronal cells. Gene. 2003/10/31 ed. 2003 Oct 30;318:113–24.

11. Citron BA, Saykally JN, Cao C, Dennis JS, Runfeldt M, Arendash GW. Transcription factor Sp1 inhibition, memory, and cytokines in a mouse model of Alzheimer’s disease. American journal of neurodegenerative disease. 2016/01/26 ed. 2015;4(2):40–8.

12. Liang H, Xiao G, Yin H, Hippenmeyer S, Horowitz JM, Ghashghaei HT. Neural development is dependent on the function of specificity protein 2 in cell cycle progression. Development (Cambridge, England). 2013/01/08 ed. 2013 Feb 1;140(3):552–61.

13. Zurkirchen L, Varum S, Giger S, Klug A, Häusel J, Bossart R, et al. Yin Yang 1 sustains biosynthetic demands during brain development in a stage-specific manner. Nature Communications. 2019 May 16;10(1):2192.
